# An innovative reconstruction of an enbloc resected composite giant chest and abdominal wall chondrosarcoma with 3D-composite mesh

**DOI:** 10.1186/s13019-024-02595-0

**Published:** 2024-03-14

**Authors:** Klein Dantis, Ramandeep Singh, Archit Goel, Brijesh Garg

**Affiliations:** 1 Department of CTVS, All India Institute of Medical Sciences (AIIMS), Bathinda, 151001 India; 2 Department of Radiodiagnosis, All India Institute of Medical Sciences, Bathinda, India; 3 All India Institute of Medical Sciences, Bathinda, India; 4 Department of Anesthesia, All India Institute of Medical Sciences, Bathinda, India

**Keywords:** Chondrosarcoma, Chest wall, Abdominal wall, Composite mesh, Reconstruction

## Abstract

**Background:**

Chest wall chondrosarcomas, although common, pose unique challenges due to their aggressive nature, rarity of abdominal wall involvement, and propensity for recurrence. We highlight the critical role of meticulous surgical planning, multidisciplinary collaboration, and innovative reconstruction techniques in achieving optimal outcomes for patients with composite giant chest and abdominal wall chondrosarcoma.

**Case Presentation:**

A 38-year-old female patient presented with progressive left chest and abdominal wall swelling for two years; on evaluation had a large lobulated lytic lesion arising from the left ninth rib, scalloping eighth and tenth ribs measuring 13.34 × 8.92 × 10.71 cm (anteroposterior/transverse/craniocaudal diameter) diagnosed with chondrosarcoma grade 2. A three-dimensional (3D) composite mesh was designed based on computed tomography using virtual surgical planning and computer-assisted design and manufacturing technology. She underwent wide local excision and reconstruction of the chest and abdominal wall with 3D-composite mesh under general anesthesia. The postoperative condition was uneventful, with no recurrence at 12 months follow-up.

**Conclusion:**

A 3D-composite mesh facilitates patient-specific, durable, and cost-effective chest and abdominal wall reconstruction.

## Introduction

Chest wall chondrosarcomas, although common, pose unique challenges due to their locally aggressive nature, the rarity of abdominal wall involvement, and the propensity for recurrence [1, 2]. A successful treatment warrants a complete surgical resection followed by reconstruction; further three-dimensional (3D) image reconstruction helps to plan complex cases and minimize intraoperative complications [3]. Our case highlights the critical role of meticulous surgical planning, multidisciplinary collaboration, and innovative reconstruction techniques in achieving optimal outcomes for patients with composite giant chest and abdominal wall chondrosarcoma.

## Case report

**Fig. 1:**
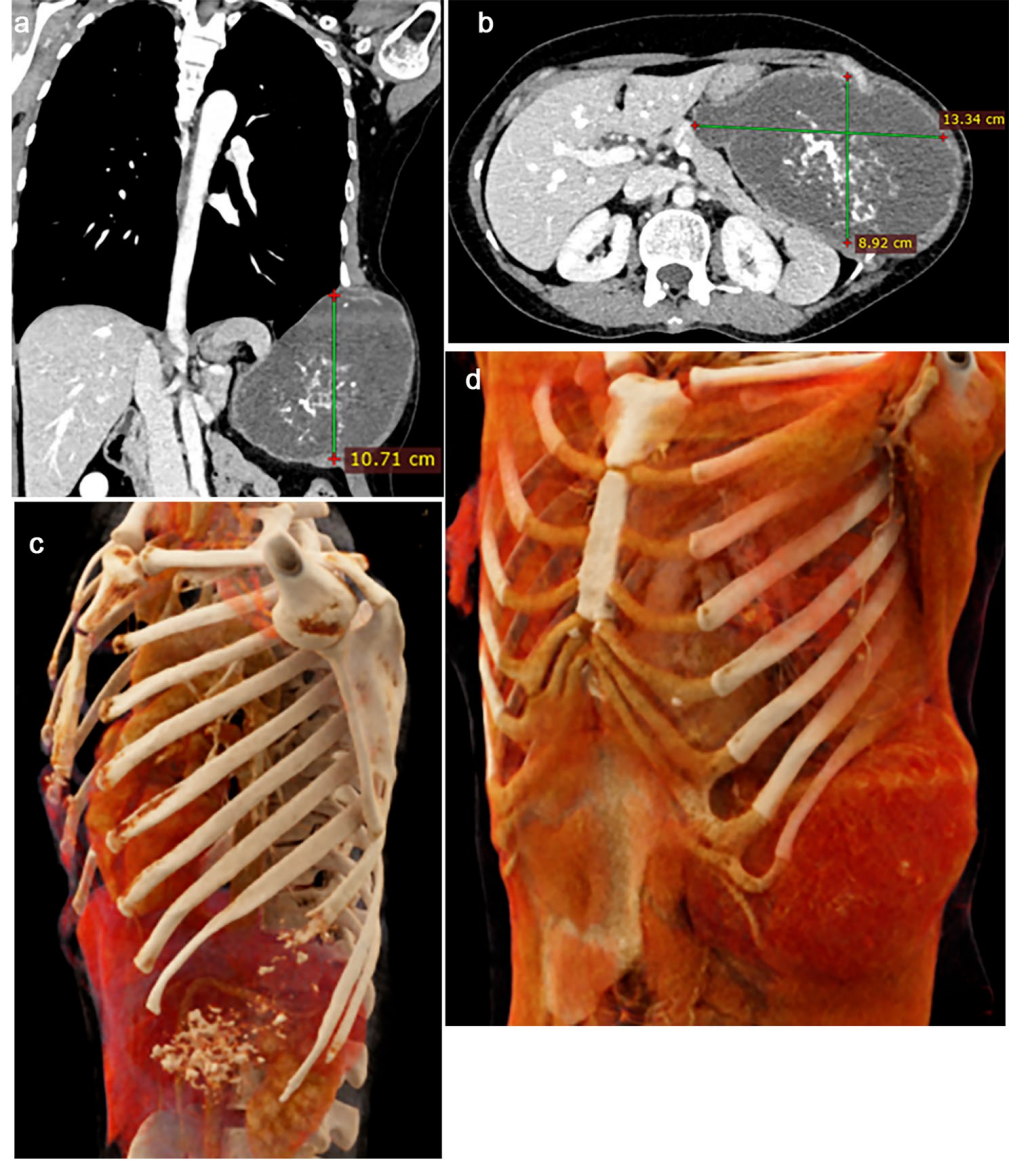
**a**: Axial view CECT thorax showing large lobulated expansile lytic lesion involving left ninth rib. **b**: Coronal view CECT thorax showing lesion with ‘ring and arc’type of calcification. **c** and **d**: 3D rendered image showing lytic destruction of the anterior body of the left ninth rib with the associated widening of intervening intercostal space by soft tissue mass and scalloping of the adjacent surface of the eighth and tenth rib

A 38-year-old female presented with progressive left chest and abdominal wall swelling for two years and had associated chest heaviness, exertional dyspnea, and intermittent fever for two months. She was hemodynamically stable with regular bowel and bladder habits. Upon examination, around 12 × 10 cm (length x width) of mass was noted, with well-defined borders and margins fixed to the chest wall. Her blood parameters, liver function tests, and kidney function tests were within normal limits. Her chest x-ray revealed homogenous opacity in the left middle and lower zone. At the same time, contrast-enhanced computed tomography of the thorax and abdomen showed a large lobulated lytic lesion involving the left ninth rib measuring 13.34 × 8.92 × 10.71 cm (anteroposterior/transverse/craniocaudal diameter). The mass abuts the diaphragm, spleen, stomach, pancreas, and overlying chest and upper abdomen muscles without invasion. It has well-defined margins and harbors a “ring and arc” type of calcification, suggesting a chondroid matrix [Fig. 1a and b]. A further 3D rendered image shows lytic destruction of the anterior body of the left ninth rib with associated widening of the intervening intercostal space by the soft tissue mass and scalloping of the adjacent surfaces of the eighth and tenth ribs [Fig. 1c and d].

The true-cut biopsy from the mass confirmed chondrosarcoma grade 2. A 3D-composite mesh was designed based on computed tomography using virtual surgical planning and computer-assisted design and manufacturing technology. Syngo. via - an integrated imaging software of Siemens Healthineers, was used to process chest and abdomen CT images acquired by a 256-slice multidetector computed tomography (MDCT) scanner [Somatom Drive (Siemens Healthineers- Erlangen, Germany)]. Cinematic volume rendering technology (cVRT) is a unique application of Syngo. via was used to get interactive 3D visualization of multi-modal imaging data. So, using cVRT, a 3D image of the chest and abdominal wall was made. Based on this, the 3D model was designed to allow precise surgical planning. Access to the chest and abdomen was through a subcostal incision.

**Fig. 2:**
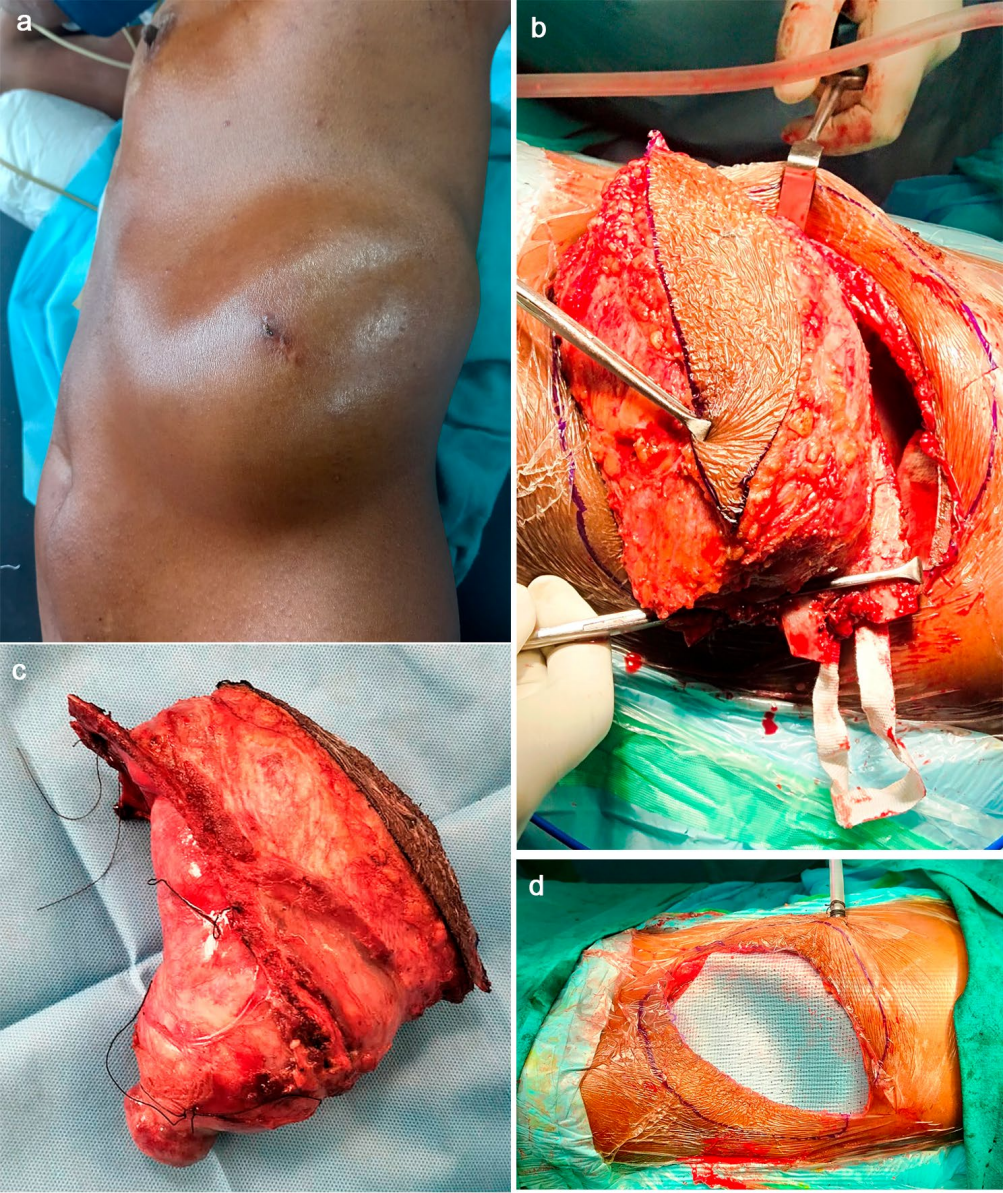
**a**: Huge chest and abdominal swelling prior to incision. **b** and **c**: Intraoperative image and complete removal of the lesion with R0 resection. **d**: 3D- composite mesh placement over the defect

**Fig. 3:**
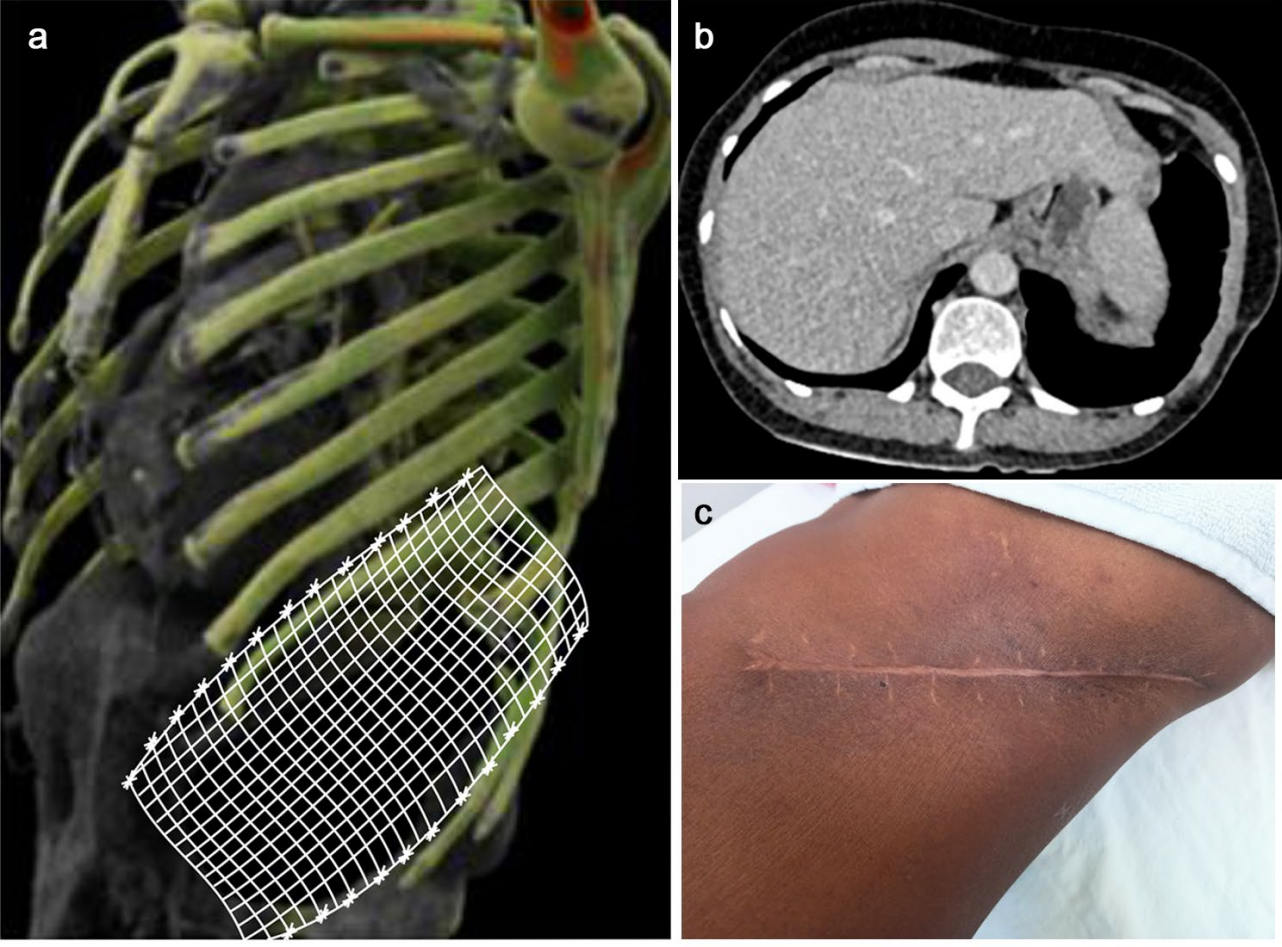
**a**: Schematic representation of 3D-composite mesh placement over the defect showed in the 3D model of the thoracic cage. **b**: Postoperative CECT thorax with no residual lesion/ recurrence. **c**: Surgical site wound healing at 12 months follow-up

Under general anesthesia and single lung ventilation, an oblique incision was made, and superior and inferior flaps were created. A 2-cm margin from the tumor site was established. Posteriorly, the eighth, ninth, and tenth ribs were resected, followed by wide local excision and excision of the tumor over the abdomen and the chest wall. At the site of the diaphragm, a 2.5 cm marginal portion of the diaphragm was also resected, and the tumor was removed entirely in toto, followed by diaphragmatic closure primarily [Fig. 2a, b and c]. Single lung ventilation was preferred in certain places during dissection to obtain an appropriate plane and avoid inadvertent parenchymal injury. A chest tube was placed, followed by reconstruction of the chest and abdominal wall. A 3D-composite mesh was placed over the defect, and edges were sutured to the intercostal muscle with prolene 3 − 0 interrupted [Figs. 2d and 3a]. Raised flaps were approximated, followed by skin closure. She was extubated after 6 h. The postoperative course was uneventful, with a drain removed on day 6, followed by discharge on day 10. Histopathology confirmed chondrosarcoma grade 2 with negative margins. Postoperative follow-up at one, three, six, and twelve months was uneventful, with no recurrence/residual lesion or chest discomfort, and the surgical site healed well by primary intention at 12 months [Fig. 3b and c].

## Discussion

The advancing technology has dramatically benefitted thoracic surgery by combining medical imaging and 3D printing, creating complex prostheses. Various chest wall reconstruction methods maximized the structural ability and aesthetic outcome, but 3D printing overcame the prosthetic effect and has gained popularity with its first report in 2013; since then, many cases have been reported in the literature [4]. However, composite giant chest and abdominal wall reconstruction with 3D-titanium implants have been reported once, with no reports to date with 3D-composite mesh [2].

Treatment for primary chest wall chondrosarcomas with defects larger than 5 cm or anterior resection involving more than three ribs primarily consists of wide surgical excision with tumor-free margins, followed by reconstruction of the defect using a prosthetic material that should be sufficiently rigid to prevent paradoxical respiratory movements, biocompatible enough to allow fibrous tissue growth without causing infections, and sufficiently radiolucent to allow radiographic follow-up [5]. The flail chest, a leading complication of wide chest wall resection, needs attention [4]. However, double-layer prolene mesh for giant chondrosarcoma of the chest wall has minimized the sandwich technique [5]. Adopting various prosthetic materials has disadvantages, including high cost, migration, infection, screw loosening, and foreign body rejection [2, 5]. At the same time, modern 3D reconstruction can provide durable and rigid construction and benefit from anatomical precision, reduced operative time, and ease of use [2, 6].

Essential factors of concern for 3D reconstruction include exothermic chemical reactions and biocompatible modulus of elasticity. Lack of exothermic reaction improves tissue healing and reduces prosthesis loosening over the long run. At the same time, the biocompatible modulus of elasticity determines the success of an implant [7]. From the surgical point of view, it should be similar to the surrounding bony tissue, avoiding stress shielding and bone resorption, thereby preventing implant loosening [8]. Though 3D reconstruction has good ergonomics, it leads to rigid reconstruction that has affected respiratory movement and postoperative quality of life [4]. However, modifications in the 3D reconstruction and Stratos titanium system with Greek wave pattern have alleviated the risk of atelectasis, improving the quality of life. However, metal fatigue with a Greek wave pattern and repeated excessive force leading to long-term implant fracture is a concern [4].

Further, a disadvantage with 3D reconstruction implant is cost, manufacturing time, and inability to alter the design intraoperatively, but 3D-composite mesh overcomes these [2]. The 3D-composite mesh comprises knitted polyethylene terephthalate filaments coated on one side with polyetherurethane. The parietal non-coated allows good tissue integration of the prosthesis. At the same time, visceral coated side polyetherurethane, because of its stability, biocompatibility, and non-adherent property, avoids or limits adhesions between the mesh and the viscera.

Our case is unique owing to the extensiveness of the defect: a large resection area of the chest and abdominal wall predisposing the patient to a paradoxical chest wall, reduced ventilatory capacity, and decreased core strength. Our decision to use 3D- composite mesh is driven by its availability, affordability, shape versatility, inert qualities over implants or titanium plates, and lack of adhering property to viscera over double-layered prolene meshes. To our knowledge, this is the first reported case managed with 3D-composite mesh. As these defects are less reported, the successful use of the technology with this reconstruction technique allows surgeons and centers to manage the case cost-effectively.

## Conclusion

A 3D-composite mesh facilitates patient-specific, durable, and cost-effective chest and abdominal wall reconstruction.

## Data Availability

The data and materials of this article are available.
